# Recurrent multi-view 6DoF pose estimation for marker-less surgical tool tracking

**DOI:** 10.1007/s11548-025-03436-8

**Published:** 2025-06-17

**Authors:** Niklas Agethen, Janis Rosskamp, Tom L. Koller, Jan Klein, Gabriel Zachmann

**Affiliations:** 1 Fraunhofer MEVIS, Max-von-Laue-Str. 2, 28359 Bremen, Germany; 2 University of Bremen, Bibliothekstraße 1, 28359 Bremen, Germany

**Keywords:** Multi-view object pose estimation, Recurrent neural networks, Marker-less tracking, Surgical navigation

## Abstract

**Purpose:**

Marker-based tracking of surgical instruments facilitates surgical navigation systems with high precision, but requires time-consuming preparation and is prone to stains or occluded markers. Deep learning promises marker-less tracking based solely on RGB videos to address these challenges. In this paper, object pose estimation is applied to surgical instrument tracking using a novel deep learning architecture.

**Methods:**

We combine pose estimation from multiple views with recurrent neural networks to better exploit temporal coherence for improved tracking. We also investigate the performance under conditions where the instrument is obscured. We enhance an existing pose (distribution) estimation pipeline by a spatio-temporal feature extractor that allows for feature incorporation along an entire sequence of frames.

**Results:**

On a synthetic dataset we achieve a mean tip error below 1.0 mm and an angle error below 0.2$^{\circ }$ using a four-camera setup. On a real dataset with four cameras we achieve an error below 3.0 mm. Under limited instrument visibility our recurrent approach can predict the tip position approximately 3 mm more precisely than the non-recurrent approach.

**Conclusion:**

Our findings on a synthetic dataset of surgical instruments demonstrate that deep-learning-based tracking using multiple cameras simultaneously can be competitive with marker-based systems. Additionally, the temporal information obtained through the architecture’s recurrent nature is advantageous when the instrument is occluded. The synthesis of multi-view and recurrence has thus been shown to enhance the reliability and usability of high-precision surgical pose estimation.

## Statements and Declarations

The project was funded by the University of Bremen Research Alliance (UBRA). The authors have no competing interests to declare that are relevant to the content of this article.

## Introduction

Surgical navigation systems facilitate a variety of applications in clinical interventions such as minimal invasive neurosurgery, stereotaxy or implant placement [1]. Combining pre-operative medical images with real-time tracking during surgery provides invaluable guidance for the surgeon and improves surgical precision, accuracy, and safety [2, 3].

Marker-based approaches achieve high precision and repeatability with errors below $1\,$mm [3]. However, the markers require to be in line-of-sight, which forces the surgeon to prevent occlusion. Furthermore, the instrument can become polluted, preventing tracking entirely and requires marker replacement. AI-based marker-less approaches could address these challenges by predicting the instrument pose from RGB images using neural networks, even with partial visibility. These techniques represent a potential future direction for surgical tracking. Significant progress has already been made for hand-object estimation [4] and multi-view pose estimation [5] for surgical instruments.

In this paper, we investigate how multi-view approaches and recurrent neural networks (RNN) can further improve the precision, reliability, and usability of surgical tracking systems. Multi-view pose estimation [6–8] leverages images from multiple cameras to enhance the accuracy and reliability of estimations compared to single-view setups [9, 10]. EpiSurfEmb [7] estimates 3D-3D correspondence distributions from single-view correspondences. CosyPose [6] uses single-view results to simultaneously optimize the positions of cameras and objects using RANSAC. The SpyroPose architecture [8] utilizes a grid-based method to compute a pose distribution. A multi-view approach is accomplished in SpyroPose by using the same grid for all views.

Additionally, recurrent architectures leverage temporal information to improve tracking performance, reducing jitter, and compensating for information loss due to partial occlusion [11, 12]. [11] applies a recurrent neural network (RNN) for temporal-information-enhanced object pose refinement, while [12] leverages temporal information for the consistency of motion within the estimation of human poses.

Our recurrent architecture incorporates convolutional GRU (ConvGRU) layers [13] into a feature extractor [14] for object pose estimation and combines the novel architecture with a multi-view approach. We investigate how these two approaches improve the tracking and in particular, how they interact with each other when combined. We conduct a study on a simulated dataset of surgical instruments with realistic hand poses. Artificial occlusion is added to analyze the behavior under partial visibility. Finally, the findings of the synthetic dataset are evaluated on a real dataset that resembles a surgical scene. All data are available online^1^. To the best of our knowledge this is the first concept to combine recurrence and multi-view for object pose estimation.

## Method

A novel recurrent multi-view architecture for 6DoF pose estimation is developed and evaluated alongside the baseline implementation. An existing multi-view pose estimation architecture is extended by recurrence to investigate the effect of temporal information and to develop a pose estimator that is more robust against object occlusion.

### Dataset creation

**Fig. 1:**
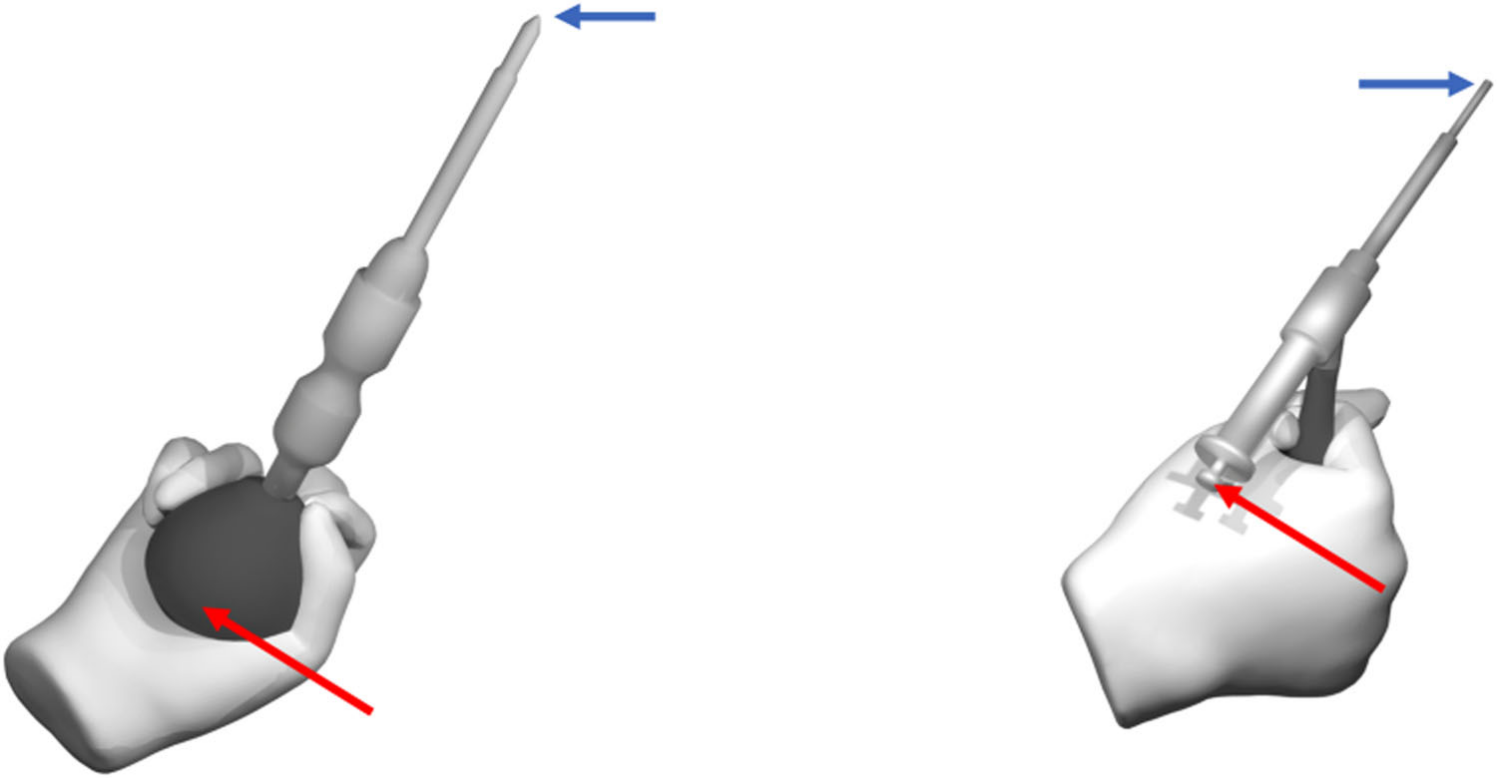
Screwdriver (left) and drill sleeve (right). The blue arrows show the tip and the red arrows the rear of the instruments. We use the line between tip and rear to measure the angle error

We create synthetic datasets featuring two medically relevant objects-a screwdriver and a drill sleeve (see Fig. 1) using BlenderProc to generate photorealistic images. Each object is grasped in 20 unique ways by a gloved hand model. Using a motion-capturing system, we record three minutes of trajectories for the instrument movement, so that the final datasets contain sequences of linearly sampled frames at 10 FPS. We also collect a real dataset using marker-based motion capture, following the approach in [15], which enables training after marker removal via inpainting.

### Pose estimation baseline

**Fig. 2:**
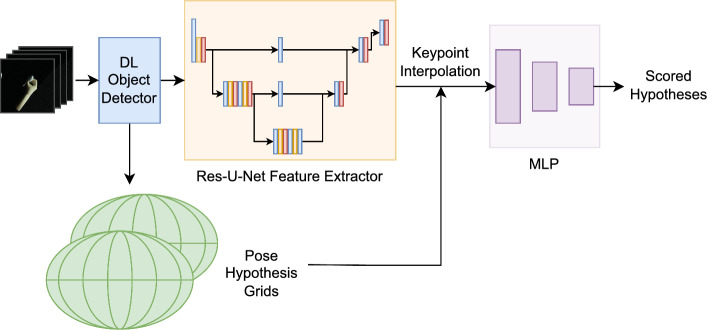
SpyroPose baseline architecture consisting of multi-level pose hypotheses grids, object detector, feature extractor and an MLP network for pose hypotheses scoring

We have selected SpyroPose as our baseline architecture due to its capabilities in multi-view pose estimation and pose distribution learning, which is particularly effective in managing object symmetries. In the following, we briefly summarize the main features. For a more detailed overview, we refer to Haugaard et al. [8]. Coarse-to-fine hierarchical grids are combined with deep-learning-based feature extraction and a multilayer perceptron (MLP)-based hypothesis scoring (see Fig. 2). A feature extraction network encodes spatial and semantic information into pixel-wise embeddings of RGB images cropped by an object detector. The feature extractor combines a U-Net [16] with a ResNet18 [17] backbone to obtain 64-dimensional features per input pixel.

The hierarchical grids differ in granularity and describe pose candidates, such that candidates from multiple levels of granularity can be obtained. For each pose candidate, represented as a grid element, keypoints are projected onto the image. These keypoints are selected using furthest-point sampling on the object’s 3D model. Interpolated keypoint features from the feature extractor output are fed into an MLP to score hypotheses by predicting unnormalized log-likelihoods. The MLP learns to differentiate between correct and incorrect pose hypotheses using the InfoNCE loss. Furthermore, SpyroPose applies importance sampling by leveraging the learned scores to focus computations on the most promising hypotheses.

### Multi-view point estimation strategy

SpyroPose generates distributions of possible poses. The pose candidate with the highest probability is selected as the final pose. We investigate additional selection methods. For surgical applications, we focus on two specific aspects: the tip position and the direction of the instrument, referred to as object angle. These features are crucial for the navigation system. The tip position is determined by using its coordinates in object space from the most likely pose candidate. The direction the instrument points is calculated by considering a second point located at the object’s rear (see Fig. 1). By focusing on these two measurements rather than directly using the 6D pose, we eliminate challenges with rotationally symmetric instruments. We’ve examined three methods to determine the final pose candidate:

- *Max Probability:* We select the 6D pose that has the highest probability as the final pose. This is the approach in SpyroPose [8].
- *Weighted Averages:* We compute the weighted average of the top n predicted poses weighted by their probabilities.
- *Grid-Based Method:* The position of the tip is represented by coordinates *x*, *y*, *z* and a probability *p*. Since errors in depth (*z*) are usually the largest, we set smaller error bounds *dx* and *dy* within the plane, and a larger bound for *dz* perpendicular to it. We create a stretched cuboid for each of the top *n* pose candidates according to these bounds. These cuboids are then arranged in a uniform grid. For grid cells where cuboids overlap, we combine their probabilities. The final 6D pose is determined by choosing the grid cell with the highest total probability.

To minimize depth ambiguity in pose estimation, we utilize images from multiple cameras. Currently, SpyroPose includes a multi-view estimation feature, where it employs the same grid across all camera views. For the recursive grid refinement, the grid cells with the highest probabilities across all cameras are selected. Essentially, SpyroPose incorporates sensor fusion directly within its neural network architecture.

**Fig. 3:**
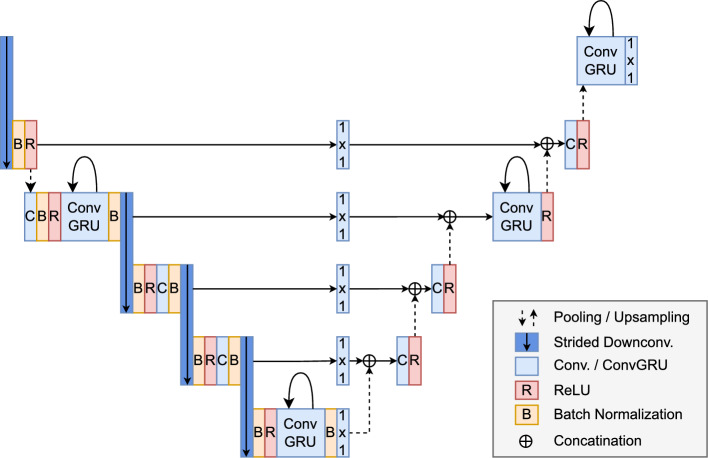
5-level Recurrent-Residual-U-Net for spatio-temporal feature extraction. ConvGRU layers replace convolutional layers on second and fifth encoder as well as third and fifth decoder level. Residual connections of the encoder are not shown to improve readability

In addition to this integrated approach, we explore late fusion, where we combine the results from individual camera views after initial pose estimations are made. To find the optimal number of views, we examine how the number of camera views affects the accuracy of the pose estimation.

### Recurrent pose estimation

Incorporating recurrence might be suitable in SpyroPose’s MLP and the feature extractor. However, extending the MLP by recurrence can be challenging as its input consists of all the feature vectors per key point for each pose candidate of a single frame. Thus, up to 512 feature vectors have to be considered for a single frame. On one hand, concatenating these features in the batch’s feature dimension leads to very large features, which is computationally expensive [13]. On the other hand, concatenating in the sequence dimension requires the recurrent layers to go back up to 512 time points per frame, which may limit the temporal processing. Furthermore, the MLP input might vary between frames due to the difference in pose hypothesis grids, which worsens the temporal consistency.

SpyroPose’s feature extractor allows for recurrence incorporation to provide sequence-enhanced features enriched by previous frames. Due to their ease of training compared to Long Short Term Memory (LSTMs) or standard RNNs, Gated Recurrent Unit (GRUs) are applied [14]. Standard GRU layers are not specifically designed for spatial inputs. They require prior feature flattening and thereby enlarge the feature vectors depending on the input’s spatial size. The introduction of ConvGRU layers promises spatio-temporal feature learning [13, 14].

The fully connected operation of standard GRU gates are replaced by convolutions in a ConvGRU, which reduces the number of weights for multi-dimensional data such as images. The convolution operation further allows focusing on regional context. Equations 1 to 4 describe the processing of a ConvGRU layer with $W$ as trainable weights, $x_t$ as input and $h_t$ as output at time *t*. The * denotes a convolution.

1 $$\begin{aligned} z_t= &   \sigma (x_t * W_{xz} +h_{t-1} * W_{hz} + b_z) \end{aligned}$$

2 $$\begin{aligned} r_t= &   \sigma (x_t * W_{xr} +h_{t-1} * W_{hr} + b_r) \end{aligned}$$

3 $$\begin{aligned} \hat{h_t}= &   tanh(x_t * W_{xh} +h_{t-1} * W_{hh} + b_h) \end{aligned}$$

4 $$\begin{aligned} h_t= &   z_t \odot h_{t-1} + (1 - z_t) \odot \hat{h_t} \end{aligned}$$

ConvGRU layers replace the convolutional layers at different stages of SpyroPose’s Residual-U-Net architecture (see Fig. 3). Randomly initialized recurrent layers are incorporated into the pretrained ResNet18 [17] encoder and decoder such that temporal information can facilitate latent representation learning as well as spatial information reconstruction. The residual nature of the encoder allows the model to ignore temporal information by using the identity connection [17].

The current implementation (RC) has been empirically shown to obtain best results compared to other variants, such as a single ConvGRU layer at the U-Net bottleneck (RB) or ConvGRU layers at every encoder and decoder level (RA) (see Table 1). Recurrence in the bottleneck seems to have a large effect as the RB and RC results are similar, in contrast to the additional GRU layers of RC. Adding a GRU layer to each level (RA) increases the number of trainable parameters by about 23 million compared to RC.

**Table 1:** Tip and angle errors of different architecture approaches obtained from the synthetic baseline dataset for the screwdriver

	Tip error (in mm)		Angle error (in degree)	
	Mean±SD	RMSD	Mean±SD	RMSAD
RB	26.32±30.51	46.00	2.39±2.99	0.0490
RB	26.32±30.51	46.00	2.39±2.99	0.0490
RA	28.30±32.13	46.77	2.51±2.68	**0**.**0445**
RC	**25**.**86**±**28**.**88**	**44**.**12**	**2**.**39**±**2**.**70**	0.0463

The best results in each category are shown in bold

RB: recurrence on bottleneck level; RA: recurrence on all levels; RC: recurrence on custom levels

### Recurrent multi-view

For the synthesis of both methods, the trained single-view recurrent models are combined with the multi-view early fusion approach. This merges spatio-temporal features with fused grids and candidate probabilities from multiple cameras.

## Experiments

The synthetic baseline training set of the conducted experiments consists of 10,000 unique scenes (120,000 total images). In each scene, a camera is randomly positioned to capture images at twelve different time points. For the test set, we create 100 scenes. In each of these, 96 images are taken from eight randomly placed cameras, capturing images at the same twelve time points. The training set lacks multi-view data, which is not required for training our neural network.

**Fig. 4:**
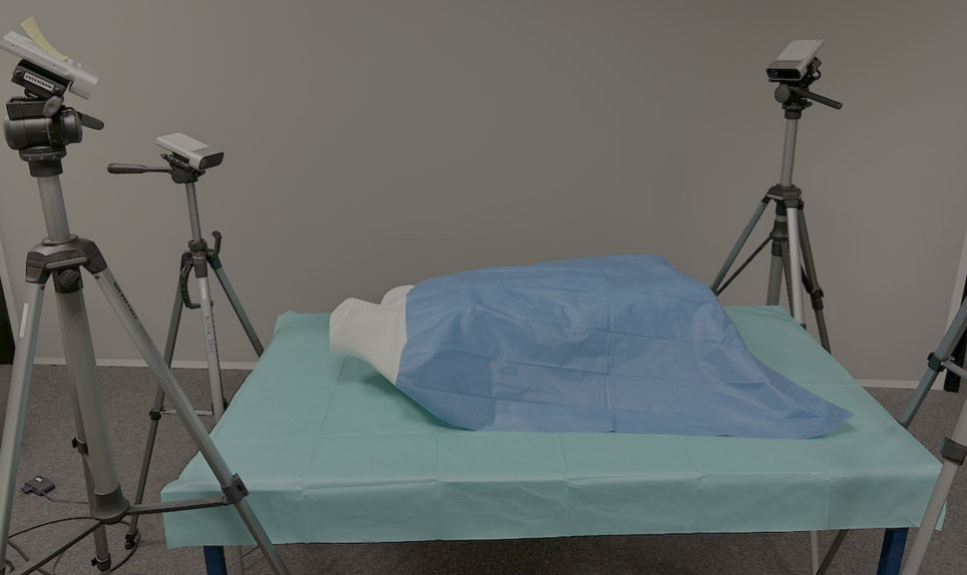
Experimental setup for collecting real-world training and test images

A second synthetic training set, referred to as the synthetic distractor dataset, contains distractor objects that are added between the sixth and ninth frame (62,400 total images). The corresponding test set applies two cameras (6,000 total images) where the view of one camera is occluded from the sixth frame onward. The real dataset consists of three scenes and a total of around 40,000 annotated images captured with four cameras at the same time. The experimental setup is shown in Fig. 4. We utilize the SpyroPose architecture with the same training parameters as those specified in [8].

### Multi-view point estimation

We evaluate the three final pose selection methods across three scenarios: i) single-view, ii) multi-view with late fusion, and iii) SpyroPose with integrated multi-view analysis, using the synthetic baseline dataset. For the multi-view approaches we use all eight cameras. The results are summarized in Table 2. For the single-view and SpyroPose multi-view scenarios, Weighted Averages performs best with a 55% reduction for multi-view in comparison with the Max Probability method of SpyroPose. For multi-view late fusion, the Grid-Based approach yields the best performance, with an error of $3.5\,$mm.

These results demonstrate that the late fusion approach is considerably less effective than using sensor fusion directly within the neural network. As indicated in Table 2, the two methods show a difference of 76%. Based on these findings we use the SpyroPose multi-view with weighted averages.

The results for different camera setups are summarized in Table 3. Our findings demonstrate a substantial improvement when employing a multi-view setup. Particularly, with six or eight views, the tip error is reduced to sub-millimeter levels, and the angle error is minimized to less than $0.15^{\circ }$. Multi-view performance on real data is lower than on the synthetic dataset. Nonetheless, performance remains strong, with single-view results matching those on synthetic data.

**Fig. 5:**
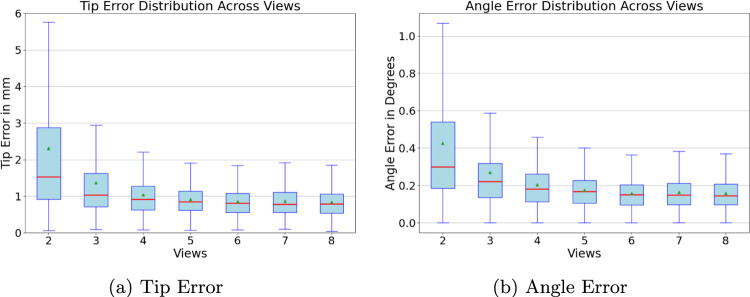
Box plot depicting the distribution of tip and angle errors in millimeters as a function of the number of cameras, ranging from 2 to 8 on the synthetic baseline dataset

Figure 5 illustrates how the accuracy of tip and angle errors is influenced by the number of camera views. The median tip error and interquartile range (IQR) decreases as the number of cameras increases, highlighting an improvement in accuracy and precision with more viewpoints. Fewer tip error outliers are observed in setups with more than four cameras, suggesting enhanced reliability. Overall, the increase in performance appears to be converging, wherefore the accuracy cannot be improved indefinitely.

**Table 2:** Tip error obtained with point estimation methods on the synthetic baseline dataset: max probability, weighted averages, and grid-based methods from the pose distribution for the screwdriver, measured in millimeters

	Single-view	Multi-view late fusion	Multi-view
Max probability	16.9	13.6	1.86
Weighted averages	15.8	5.8	0.83
Grid-based	18.3	3.5	2.4

**Table 3:** Influence of number of views on tip error and angle error for the synthetic baseline and the real datasets

	Views	Screwdriver		Drill sleeve	
		Tip error (mm)	Angle error ($^{\circ }$)	Tip error (mm)	Angle error ($^{\circ }$)
Synthetic	1	15.80	1.43	11.83	1.02
	2	2.37	0.47	1.90	0.47
	4	1.04	0.20	0.75	0.18
	6	0.86	0.16	0.57	0.14
	8	0.83	0.15	0.55	0.13
Real	1	11.50	1.87	16.05	2.05
	2	4.23	0.65	4.15	0.69
	4	2.85	0.44	2.64	0.53

### Recurrent single-view

Temporal information is expected to be particularly useful when visual information is limited, e.g., due to object occlusion [11]. In order to investigate the recurrent performance under these circumstances, experiments with artificial occlusion through a checkerboard overlay are conducted using the synthetic baseline dataset. Occlusion is randomly applied to 50% of the frames in the second half of each sequence to ensure that objects are visible at the beginning. Furthermore, the checkerboard pattern is added with a random offset. For better comparability, the test set frames are identical across different model evaluations. The models are trained and evaluated with and without artificially occluded frames. Evaluation metrics include the tip positional error and object angle error as well as metrics measuring the smoothness of the predicted trajectories, namely root mean squared deviation (RMSDs) and root mean squared angular deviation (RMSAD). The RMSD and RMSAD measure the deviation of the tip position and object angle between subsequent frames. Due to the actual movement of the instrument between frames, the RMSD and RMSAD of a smooth trajectory prediction are not expected to be zero but close to the ground truth.

The following models are evaluated as shown in Table 4:

- Non-recurrent baseline (NRB) trained without occlusion
- Non-recurrent model trained with occlusion (NRO)
- Non-recurrent model trained with sequential batch sampling and occlusion (NRSBO)
- Recurrent baseline (RB) trained without occlusion
- Recurrent model trained with occlusion (RO)

The baseline experiment (NRB) applies random frame sampling and data augmentation as per [8] to the training set without occlusion. The baseline achieves the best results for the screwdriver on the non-occluded test set with a mean tip error of 15.80 mm and a mean angle error of 1.43$^{\circ }$ . The mean results for the drill sleeve are 11.83 mm and 1.02$^{\circ }$. The NRO model predicts the instruments’ pose similarly well as the baseline. To investigate the effect of batch variance, the non-recurrent model (NRSBO) is trained with occlusion and the same sequence batch sampling as the recurrent models, where batches consist of entire sequences. The shrinkage in batch variance has a severe impact on the evaluation metrics for both instruments. The experiments with the recurrent architecture achieve similar results as the NRSBO model, thus all metrics are worse than the other non-recurrent approaches.

On the occluded dataset, the recurrent architecture improves the performance. Models trained without occlusion have considerably larger errors when applied to an occluded test set, as not being faced with similar data during training. Also for the models trained with occlusion the metrics drop but less severely. The non-recurrent model (NRO) predicts the tip with a mean error of 29.46 mm and 22.79 mm. The recurrent approach (RO) is able to outperform the non-recurrent in all metrics with a mean tip error for the screwdriver of 25.86 mm and 19.57 mm for the drill sleeve. Similarly, the angle error and trajectory smoothness metrics improve.

**Table 4:** Single-view results of the synthetic baseline test set with and without checkerboard occlusion separated by surgical instruments

		Test set without occlusion		Test set with occlusion	
		Mean±SD	RMSD / RMSAD	Mean±SD	RMSD / RMSAD
*Tip error (in mm)*					
Screw driver	NRB	**15.80±12.80**	**23.35**	73.48±170.56	204.20
	NRO	16.74±13.46	24.20	29.46±51.62	64.70
	NRSBO	20.71±18.17	27.60	37.40±63.15	77.01
	RB	19.51±16.41	25.92	64.47±135.26	164.86
	RO	19.37±15.72	26.52	**25.86±28.88**	**44**.**12**
Drill sleeve	NRB	11.83±9.87	**19**.**57**	58.30±147.46	167.26
	NRO	**11.57±9.59**	19.95	22.79±42.90	54.72
	NRSBO	12.44±10.70	20.57	25.69±54.11	62.46
	RB	12.66±11.52	20.81	50.58±126.67	139.93
	RO	12.74±11.62	21.01	**19.57±26.40**	**38**.**90**
*Angle Error (in degree)*					
Screw driver	NRB	**1.43±1.51**	**0**.**0103**	9.55±25.80	0.4549
	NRO	1.50±1.54	0.0261	3.48±9.58	0.1312
	NRSBO	1.91±1.90	0.0318	4.83±12.76	0.1772
	RB	1.84±2.00	0.0288	8.07±22.82	0.3643
	RO	1.81±1.79	0.0296	**2.39±2.70**	**0**.**0463**
Drill sleeve	NRB	1.02±1.22	**0**.**0220**	7.19±20.62	0.3372
	NRO	**1.00±1.00**	0.0223	2.65±8.26	0.1095
	NRSBO	1.06±1.00	0.0232	3.30±10.60	0.1417
	RB	1.09±1.12	0.0235	4.59±12.73	0.1902
	RO	1.07±1.04	0.0229	**1.64±1.98**	**0**.**0405**

The best results in each category are shown in bold

**Fig. 6:**
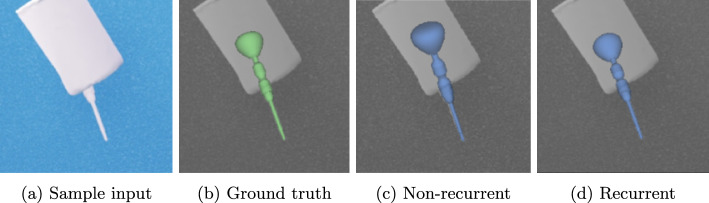
Sample from the distractor test set depicting the occluded screwdriver

Figure 6 depicts a screwdriver sample with distractor occlusion, which demonstrates the recurrent architecture’s strength of facilitating previous frames in case of ambiguous poses. While the non-recurrent model predicts a plausible yet false angle of the occluded instrument, the recurrent model can leverage temporal information to resolve the ambiguity.

**Fig. 7:**
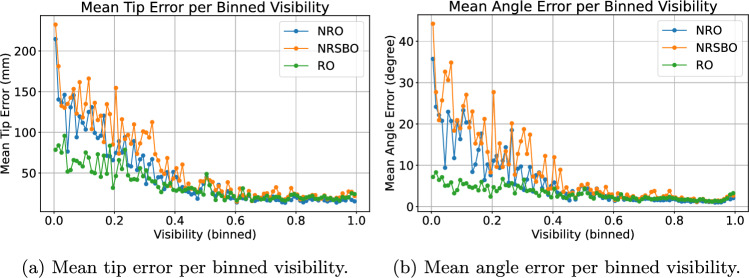
Mean tip and angle error of recurrent and non-recurrent models applied to the screwdriver test set for binned visibilities with each bin of size 1%

Figure 7 highlights the beneficial effect of recurrence regarding tip and angle error with respect to object visibility. The visibility is measured by the percentage of visible surface pixels considering occlusion by scene objects, hands or the artificial checkerboard compared to the visible pixels without any occlusion. The heavier the instrument is occluded, the better is the recurrent prediction compared to the non-recurrent. In the interval between 20% and 40% visibility, the recurrent architecture achieves a tip error of 44.70±12.10 mm and an angle error of 4.29±1.15$^{\circ }$, compared to 59.58±18.38 mm and 8.27±3.83$^{\circ }$ for the non-recurrent architecture.

**Fig. 8:**
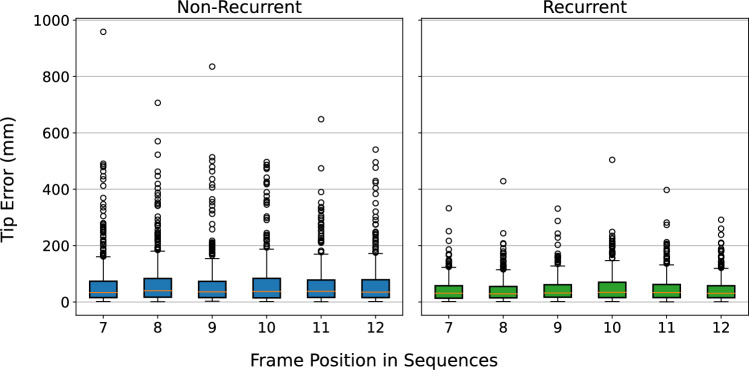
Distribution of the tip error of the non-recurrent and recurrent model for the screwdriver test set with checkerboard occlusion considering occluded frames only

Figure 8 shows the screwdriver tip error distribution for the non-recurrent and recurrent model. Only the latter half of the sequence is displayed, where all frames are occluded with the checkerboard pattern. The lack of considerable differences is expected in the non-recurrent approach, while the result of the recurrent approach indicates that the temporal receptive field covers the six occluded frames over the period of 0.6 s and suggests experiments with longer sequence lengths.

### Recurrent multi-view

Recurrent multi-view experiments combine both methods by processing a batch of frames from multiple cameras of an entire sequence. The experiments use the synthetic baseline dataset with two out of eight cameras. The results resemble the findings from the single-view experiments and are presented in Table 5. Without occlusion, the benefit of recurrence seems negligible, and temporal information cannot compensate for the lower variance in training data. In general, the results of the different models do not deviate considerably across all metrics. For the screwdriver the best result is achieved by the non-recurrent baseline (NRB) with a mean tip error of 2.37±1.45 mm, for the drill sleeve the recurrent model (RO) achieves the lowest mean tip error with 1.87±1.28 mm. As recurrence does not considerably improve the results for two cameras and the effect of recurrence is expected to decrease with increasing number of views, experiments with more cameras are not conducted.

**Table 5:** Multi-view results of the synthetic baseline test set with multi-view setup using two cameras

		Test set without occlusion		Test set with occlusion	
		Mean±SD	RMSD / RMSAD	Mean±SD	RMSD / RMSAD
*Tip error (in mm)*					
Screw driver	NRB	**2.37±1.45**	**16.52**	11.86±43.95	42.44
	NRO	2.42±1.44	16.57	**4.39±11.11**	20.97
	NRSBO	2.49±1.48	16.63	6.32±16.97	26.17
	RO	2.56±1.50	16.56	**4.52±7.64**	**19**.**93**
Drill sleeve	NRB	**1.90±1.26**	**14**.**53**	7.84±33.04	33.23
	NRO	1.92±1.48	14.55	4.07±10.99	19.03
	NRSBO	1.92±1.35	14.56	4.14±17.87	21.06
	RO	**1.87±1.28**	14.57	**3.92±8.84**	**18**.**47**
*Angle error (in degree)*					
Screw driver	NRB	**0.47±0.28**	**0**.**0167**	2.29±11.73	0.1104
	NRO	0.50±0.29	0.0182	0.73±1.67	0.0283
	NRSBO	0.50±0.29	0.0174	1.15±3.47	0.0504
	RO	0.52±0.30	0.0174	**0.71±0.79**	**0**.**0218**
Drill sleeve	NRB	0.47±0.39	0.0166	2.08±10.97	0.1168
	NRO	0.49±0.48	**0**.**0160**	0.86±2.73	0.0347
	NRSBO	0.48±0.38	0.0168	0.91±4.27	0.0437
	RO	**0.47±0.38**	0.0167	**0.64±0.73**	**0**.**0213**

The best results in each category are shown in bold

**Fig. 9:**
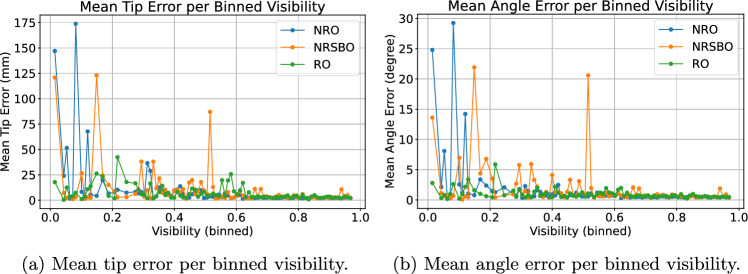
Mean tip and angle error of the screwdriver per binned visibility of recurrent and non-recurrent models applied to the checkerboard occlusion test set in a setup with two cameras. Visibility is measured as the average surface visibility across both views

When adding artificial checkerboard occlusion to the test set, the recurrent results are able to outperform the non-recurrent in all metrics but the mean tip error of the screwdriver (NRO: 4.39±11.11 mm, RO: 4.52±7.64 mm). For the drill sleeve, the RO model achieves the best tip error of 3.92±8.84 mm, while the NRO model error is 4.07±10.99 mm. The occlusion pattern is randomly added to both views of the test set sequences. In case of low mean instrument visibility across both views, the recurrent model is able to improve upon the non-recurrent (see Fig. 9). In the interval between 20% and 40% visibility, the mean tip error of the RO model is about 3 mm better than the non-recurrent (6.44±4.07 mm and 9.65±8.52 mm).

**Table 6:** Multi-view results of the synthetic test set with distractor, where one of two cameras has an occluded view toward the instrument

		Test set with distractor			
		Tip error (mm)		Angle error (degree)	
		Mean±SD	RMSD	Mean±SD	RMSAD
Screw driver	NRD	3.26±7.24	16.61	0.62±1.19	0.0188
	RD	**3.07±4.23**	**15**.**91**	**0.59±0.57**	**0**.**0165**
Drill sleeve	NRD	2.73±4.93	14.22	0.55±0.62	0.0164
	RD	**2.45±2.96**	**14**.**08**	**0.51±0.47**	**0**.**0161**

The best results in each category are shown in bold

**Table 7:** Results of the non-recurrent (NRR) and recurrent (RR) model for the real test set with two cameras

		Real test set	
		Mean tip error (mm)	Mean angle error (degree)
Screw driver	NRR	4.23	**0**.**65**
	RR	**3**.**94**	**0**.**65**
Drill Sleeve	NRR	**4**.**15**	**0**.**69**
	RR	4.20	0.90

The best results in each category are shown in bold

To examine the beneficial effect of temporal information in a more realistic occlusion setting, models are trained on the synthetic distractor and the real training set and evaluated on the respective test set containing two cameras. As shown in Table 6, the results of the distractor test set resemble the checkerboard occlusion results, where the recurrent (RD) outperforms the non-recurrent (NRD) model on all metrics. In contrast to the checkerboard occlusion, the distractor test set contains only sequences with one of two cameras with an occluded view toward the target instrument, which explains the slightly better result. The performance on the real test set is shown in Table 7. The recurrent (RR) model achieves slightly better results for the screwdriver (mean tip error of 3.94 mm), while the non-recurrent the slightly better for the drill sleeve (4.15 mm).

## Discussion

Our experiments emphasize that a multi-view setup is necessary to achieve surgically required precision. In our analysis of camera configurations, it is evident that increasing the number of cameras generally leads to better results. However, a high number of cameras might not always be practical in real-world clinical settings due to space, cost, or logistical constraints. When evaluating real data, we observe that pose estimation performance is generally lower compared to the synthetic dataset. This discrepancy may stem from labeling inaccuracies, despite careful annotation. Additionally, the real dataset may present inherently greater challenges due to the complexity and variability of real-world conditions. Further investigation is needed to fully understand and address these differences. Overall, multi-view configurations, particularly those with four or more cameras, show potential for providing tip and angle estimates that approach the requirements for clinical applications.

Still, the trained model’s performance degrades with limited object visibility. The novel recurrent architecture is able to improve the pose prediction robustness under these circumstances. The single-view results obtained on the synthetic test set with checkerboard occlusion demonstrate that the recurrent architecture is capable of leveraging temporal information to improve the pose prediction. However, the non-occluded precision cannot be obtained. Without occlusion, the recurrent architecture performs worse due to the lower batch variance during training. In a two-camera setting, the positive effect of recurrence can be confirmed on the synthetic test set with more realistic occlusion from distractor objects that take into account occlusion dependencies across frames and views. Still, the likelihood that at least one camera has good visibility is increased for a multi-view setup and the described angle ambiguity is less likely. Although the recurrence benefit appears to be lower in the real dataset, the less prominent occlusion of this dataset needs to be considered. Further exploring occlusion in a realistic surgical environment is a potential future direction.

With respect to the clinical application, the recurrent architecture can enhance the navigation system’s usability as instrument poses can still be predicted under heavy occlusion. For critical situations during the surgery, the accuracy of an occluded instrument remains insufficient, such that the clinician has to ensure clear line-of-sight for the cameras to obtain high pose prediction precision. Furthermore, the recurrent architecture might be of interest in other computer vision tasks where occlusion robustness is critical and precision requirements are lower.

### Future work

The recurrent architecture’s dependency toward batch variance could be tackled in another future work, as this has been shown as a limitation of the recurrent models. Possible directions could be advanced augmentations, longer training with more training data, and architectural changes, such as replacing batch normalization layers. Furthermore, the applied object detector could be investigated in a recurrent setup to ensure its applicability under heavy object occlusion, e.g., by incorporating recurrence.

## Conclusion

We applied marker-less 6DoF pose distribution learning to instruments commonly used in surgical navigation systems. Using synthetic and real datasets of two realistic surgical instruments, our experiments demonstrate the true potential of marker-less multi-view pose estimation. While single-camera tracking yields a mean tip error above 10 mm and a mean angle error above 1$^{\circ }$, the multi-camera setup achieves sub-millimeter and sub-degree accuracy. These trends are mirrored in experiments on a real dataset, where single-camera tracking similarly results in tip errors exceeding 10 mm, while a four-camera configuration reduces this to 3.0 mm or less.

By extending the deep-learning-based pose estimation pipeline with a recurrent feature extractor, we are able to exploit the temporal information of video sequences. This temporal information has been shown particularly beneficial when the frame’s visual information is limited, e.g., due to instrument occlusion. Even under heavy occlusion where only between 20% and 40% of the instrument surface is visible, a setup of only two cameras and our novel recurrent architecture enhances the mean tip error by approximately 3 mm compared to the non-recurrent model. The recurrent architecture thus serves as a prototype for incorporating temporal information into 6DoF pose distribution learning and improves the reliability and usability of surgical navigation systems.

## Footnotes

1 https://cgvr.informatik.uni-bremen.de/research/ai_surgical_navigation/.
